# What Can We Learn From Qualitative Impact Evaluations About the Effectiveness of Lobby and Advocacy? A Meta-Evaluation of Dutch aid Programmes and Assessment Tool

**DOI:** 10.1177/0193841X251314731

**Published:** 2025-01-11

**Authors:** Hugh Sharma Waddington, Hikari Umezawa, Howard White

**Affiliations:** 1 413371 London School of Hygiene and Tropical Medicine and London International Development Centre, London, UK; 2 591369 The Campbell Collaboration, Philadelphia, PA, USA; 3 193563 Global Development Network, Lanzhou University and Director of Evaluation, New Delhi, India

**Keywords:** impact evaluation, contribution analysis, qualitative evaluation, evidence synthesis, aid effectiveness, critical appraisal

## Abstract

Official development agencies are increasingly supporting civil society lobby and advocacy (L&A) to address poverty and human rights. However, there are challenges in evaluating L&A. As programme objectives are often to change policies or practices in a single institution like a Government Ministry, L&A programmes are often not amenable to large-n impact evaluation methods. They often work in strategic partnerships to foster change; hence, contribution may be a more relevant evaluation question than attribution. Small-n qualitative approaches are available to measure the effectiveness of L&A which use the theory of change as their analytical framework. We conducted a meta-evaluation of 36 evaluations of multi-component international programmes to support civil society L&A across Asia, Africa and Latin America, comprising the majority of programmatic support from one international donor. We assessed the confidence in causal claims in the evaluations using a new tool that we developed. Assessments of the contribution of the programmes to the changes in outcomes were not provided in many of the evaluations, nor were predictable sources of bias addressed. Given that L&A programmes are likely to adopt an influencing approach where many different inside-track and outside-track engagement objectives, opportunities and strategies are attempted, many of which might be expected to fail, there appeared to be a clear bias in the evaluations towards reporting outcomes that were achieved, ignoring those that were not. We provide guidance on how to improve the design, conduct and reporting of small-n qualitative evaluations of aid effectiveness.

## Introduction

There is increased emphasis in international development circles, and official development assistance programmes more specifically, on tackling poverty through its root causes at a societal level, by addressing imbalances in decision making power and institutionalised inequality (World Bank, 2003, 2017). There is therefore great interest among governments, civil society organisations (CSOs) and researchers in evaluating the effectiveness – that is, making causal inferences to outcomes of interest – of programmes that aim to address societal level problems. The effectiveness of interventions to address many societal problems may be evaluated using large-n experimental and quasi-experimental counterfactual methods, which rely on sufficient numbers of treatment units and participants for statistical inference. There are many relevant examples of these evaluations of programmes that address specific constraints faced by individuals and communities (Sabet & Brown, 2018), or in specific sectors like governance (Phillips et al., 2017), microfinance (Duvendack & Mader, 2019) or water and sanitation (Chirgwin et al., 2021) among others.

However, evaluating effectiveness of programmes that aim to hold governments and other organisations accountable, like supporting the lobby and advocacy (L&A) efforts of CSOs, poses challenges. Where programmes are aiming to influence decision making in a particular institution, such as support to lobby and advocacy (L&A) that aims to change policy in a Presidency or a Government Ministry, small-n qualitative impact evaluation approaches are needed. These methods evaluate effectiveness using factual theory-based inference, which articulates the causal pathways thought to operate to produce outputs and outcomes from the programme inputs and activities.

A large number of methods exist to conduct such evaluations, but there is limited agreement on what constitutes credible causal inference and how to design, conduct and report them appropriately. In these circumstances, causal inference requires incorporating some method of testing for alternative causal claims, such as those operating because of other programmes happening or pre-existing capabilities of CSOs. Providing a transparent decision framework on which confidence assessments can be based would be useful, including – or perhaps especially – where sponsors have pre-existing beliefs that limit the confidence in the outcomes caused by the L&A work but are seeking provisional evidence that either supports or argues against continued funding, or on how to improve its effectiveness.

In this paper, we present results of a meta-evaluation of 36 programme evaluations of Dutch government support to CSO lobby and advocacy. Meta-evaluation aims to provide evidence on the confidence in the evaluations conducted on a particular topic, and therefore whether findings are credible (HM Treasury, 2020). A primary component of meta-evaluation, and evidence synthesis more generally, is to assess the design, conduct and reporting of the evaluation method used, also called critical appraisal. Many assessment toolkits exist to appraise and synthesise findings from traditional impact evaluation methods like randomised controlled trials (RCTs) and quasi-experiments (e.g. Sterne et al., 2019; Wells et al., undated) or questions relating to implementation processes in qualitative studies (Montgomery et al., 2013), but we are not aware of any synthesis studies or tools that aim to assess the credibility of causal inference in the qualitative impact evaluation literature.

We assessed the credibility of the evidence on effectiveness and the programmes’ achievements, in order to provide guidance about methods for evaluating support to L&A. We applied a new assessment tool for small-n qualitative impact evaluations to assess the confidence in the findings about effectiveness in the 36 programme evaluations. We also harvested outcomes from these studies, assessed whether they were reportedly positive or negative changes and what strength of contribution had been assigned to them by the authors.^1^

The paper is structured as follows. The next section overviews the programme evaluations and the complexities in evaluation L&A. Section 3 presents the data collection approach and confidence assessment tool. Section 4 presents the results of the assessments. Section 5 discusses the findings, and Section 6 concludes with implications for evaluation practice.

## Background

In 2016–2020, the Dutch Ministry of Foreign Affairs (MFA) provided Euros1.2 billion in official development assistance for L&A in low- and middle-income countries (L&MICs) and at global levels. This support was provided through the Dialogue and Dissent (D&D) Programme and the Sexual and Reproductive Health and Rights (SRHR) Partnership Fund. All programmes aimed to support civil society organisation (CSO) engagement in communities and policy-level debates with government or private sector actors and thus improve attitudes, policies and policy implementation (Kamstra, 2017). They did so by supporting the capacity of grassroots organisations and national CSOs to promote leadership among Southern partners in undertaking L&A, in many cases by catalysing strategic partnerships for dialogue and dissent to influence community leaders, governments and other powerful bodies (including those operating in the private sector), to provide voice and funding for projects to improve accountability for community and policy change, with the goal of promoting inclusive laws, policies and practices. Programmes with SRHR objectives also aimed to improve service delivery and use.

Thirty-two of the MFA programmes were independently evaluated. Existing methods agree that it is particularly challenging to evaluate the effectiveness of L&A. Because L&A programmes necessarily operate as partnerships, often aiming to create a critical mass of influencing with multiple external actors involved in the various policy areas of interest, it is often appropriate to think about contribution, rather than attribution, when assessing their effectiveness. Evaluations of L&A must necessarily use a broader range of techniques than has been commonplace in impact evaluation, particularly those that can be applied to small-n cases. The methods available in the evaluation toolkit for small-n evaluation include contribution analysis (CA) (Mayne, 2020), method for the assessment of programmes and projects (MAPP) (Neubert, 1998), most significant change (MSC) (Davies & Dart, 2005), outcome harvesting (OH) (Wilson-Grau, 2019), the general elimination methodology (Scriven, 2008), process tracing (Ford et al., 1989) and the qualitative impact protocol (QuIP) (Copestake et al., 2019), among others.^2^ These methods usually use qualitative data collection and analysis approaches, including key informant interviews (KIIs), focus group discussions (FGDs) and triangulation of evidence in order to substantiate causal claims.

Van Wessel (2018) argues that the disadvantage of small-n qualitative approaches like MSC and OH are that they are highly intervention-focused, paying scant attention to the role of the context in affecting change (i.e. likely to lead to ‘self-serving’ or ‘intervention-centric’ bias). This suggests a strong understanding of the context in which the intervention strategy was implemented is an important prerequisite for evaluating causal claims. She also argues for an appreciation of systems dynamics including non-linearity (e.g. interaction effects and feedback loops) and the need to understand that L&A strategy planning is only partly plannable and necessarily adaptive. For example, the programmes reviewed here aimed to achieve changes in policies at national and international levels, involved multiple interacting local and global partners and had multiple objectives and sub-projects operating in different geographies, which occurred at the same time as other programmes or factors which could affect outcomes of interest. The implication is that assessing relative contribution is an important component of any approach to evaluating effectiveness of L&A, although it is very challenging to do.

Barret et al. (2016) emphasise the role of the theory of change (ToC) in evaluating the contribution of L&A to policy outcomes. They suggest that it is useful to limit the number of outcomes being evaluated, so as to balance accuracy of the approach with resources available for evaluation. We adopted a ToC-based approach, going beyond Barret et al. (2016), to discuss the basis for the causal claims in the ToCs. A ToC has two important roles in an evaluation: (1) to frame the evaluation, and so identify relevant evaluation questions; and (2) to test the theory and so conclude why or why not the intervention works. While relationships in a ToC are probabilistic, the final steps to policy change (e.g. more equitable service delivery) are potentially the most difficult to achieve due to the wide range of variables that affect decision making about policies and their implementation. Thus, supporting L&A activities by CSOs increases the likelihood of policy effects but does not guarantee them. ToCs may show necessary conditions, but these are rarely likely to be sufficient (Davies, 2018) or may be an Insufficient but Necessary component of a condition that is itself Unnecessary but Sufficient (INUS) for the effect to occur (Mackie, 1974).

In addition, what makes L&A different from other intervention strategies is that only a small proportion of advocacy projects may expect to achieve such success, so supporting L&A is analogous to venture capital in which most investments fail, but if they succeed, the pay-off is potentially high (Teles & Schmitt, 2011). The implication is that failure to achieve policy change or implementation is not necessarily a failure of the project being evaluated. Even where an explicit theory of change for particular strategies or tactics is difficult to specify ex-ante, due to the need for an adaptive strategy to support L&A, having an overarching programme theory of change that links desired outcomes with key activities – for example, the approach to building and supporting L&A capacity – is a useful starting point, and indeed was present in most cases we reviewed.

Another explanation of failures is that they can arise due to the quality of the supported L&A activities. Inside-track and outside-track advocacy may counter one another if they are not coordinated. For example, inside-track advocacy requires a relationship with some trust, which public (outside-track) advocacy mitigates against. On the other hand, credibility with the public and other campaigners may be compromised if an organisation is seen as too close to government or private sector bodies. Outside-track advocacy may cause positions of powerful people to become further entrenched, lead to prison sentences for campaigners or cause the organisation to lose the right to funding, or even to exist legally. That, in turn, indicates the importance of evaluating the effectiveness of building capacity of CSOs, or, more generally, immediate outcomes further back along the causal pathway, which are therefore under greater control by implementers, such as supporting partnerships and L&A practice.

But failure to achieve distal outcomes like policy changes may also arise due to external factors that inhibit the project from achieving its intended effects. For example, the project may be well-implemented with activities appropriately adjusted to the context and implemented with the right expertise (i.e. fidelity), but other factors (e.g. political instability, weak state capability and external shocks) inhibit the project from having meaningful effects on policy change.

Finally, there are a number of well-known biases that affect all types of evaluation such as bias in selection of cases, which limits the representativeness of the data available, and biases affecting the quality of data collection, such as when information is reported by study participants, as in the case of social desirability bias. In the special case when causal relationships are inferred qualitatively by participants in small-n impact evaluations, problems arise due to the systematic tendency of people to misrepresent the strength of a causal relationship, positional biases such as the ‘fundamental error of attribution’ or self-importance bias, all of which mean that people are more likely to ascribe changes to individuals and projects rather than contextual factors like general social forces (White & Phillips, 2012).

## Approach

In our assessment, the minimum criteria for confidence in an evaluation, of whatever type, are that it (1) clearly defines an appropriate study design, (2) adequately conducts it and (3) reports how the study was designed and conducted sufficiently for external replications. However, we believe that an evaluation of the effectiveness of an intervention strategy should go further by also (4) attempting to address possible sources of bias in the data being collected with reference to an explicit theory of change, (5) addressing sources of bias in causal claims being made through a sampling strategy that includes informed independent sources and other known sources of bias in reporting and (6) incorporating approaches that can measure and validate contribution to outcomes achieved relative to other programmes and relevant contextual factors. In other words, if an evaluation satisfies items (1) through (6), the findings can potentially be assessed as being of ‘high confidence’.

The approach we used drew evaluation quality criteria from the Policy and Operations Evaluation Department (IOB) (2022), and best practices in critical appraisal assessment (e.g. Jüni et al., 1999). We developed and piloted coding tools that aimed to assess the methods used to evaluate the strength of evidence on effectiveness and to harvest outcomes contained in the evaluations. This assessment tool aimed to evaluate those dimensions considered important in confidence assessment frameworks, including the substantiation of findings, application of appropriate methodology, accessible, appropriate and inclusive reporting, and analysis of context (Pollard & Forss, 2022).

The coding form, containing 65 signalling questions (Supplement 1), was developed collaboratively with stakeholders drawing on existing assessment approaches for qualitative evaluation, including the Critical Skills Appraisal Programme (CASP, 2018) and White et al. (2021). We drew on relevant guidance for design and conduct of small-n qualitative approaches, listed above and consulted existing literature on L&A evaluation to inform the content of the coding forms and our assessment of the evaluation design in the included studies (e.g. Barret et al., 2016; Teles & Schmitt, 2011; Van Wessel, 2018 ).^3^ The main points to note are: (1) we included all items related to effectiveness from the IOB (2022) evaluation quality criteria, and some additional items from those guidelines which we thought important for assessing effectiveness, for example, intervention and outcome descriptions; and (2) we elaborated the assessment criteria by breaking them down into several sub-questions, for example, by listing possible sources of bias and how they may be addressed. Our piloting suggested that breaking down the criteria in this way reduced the need for judgement in applying criteria, thus increasing the reliability and validity of the initial coding. The development and the tool itself are discussed in detail elsewhere (Sharma Waddington et al., 2023).

We considered the methods used to evaluate effectiveness and whether they were conducted appropriately. For example, OH and MSC should include substantiation of outcomes, stories and contributions through additional data collection. CA involves the chain of expected outcomes being shown as having occurred, and other influencing factors ruled out or relative contribution recognised. Stakeholder views and experiences can help shed valuable light on causal processes. The data extraction form covered questions which may apply regardless of the methods used, though the degree to which a specific question applies may vary. Evaluation approaches should be transparent in the conduct and reporting of methods and results, which is why conduct and reporting are a major focus of the data collection form.

We also considered the selection of people from whom data were collected, and the weight given to different voices. Hence, a first question for the methodology was what approach was used to evaluate effectiveness, whether OH, CA or other? Regarding conduct, a question was whether the evaluation conducted a stakeholder mapping, if so, what was the source of the data for that mapping, and whether the sample of people spoken to was drawn from across the stakeholder map? We attempted to investigate ‘insider bias’ whereby evaluations speak to people inside the project or closely connected to it, but not to those outside the project or those who might even be actively excluded. Evaluation teams may not speak to politicians, religious leaders, trade unionists, traditional leaders, such as chiefs and headmen or women, and journalists, even though these are all important groups of opinion leaders who may be well-informed regarding the issues at hand.

Another example is whether the evaluation measured CSO capacity, and if so, how and what method was used to assess whether any changes resulted from project activities, either in aggregate or by component? We therefore also investigated whether assessments of capacity, either that already existing or built and supported through the programme, were made.

We also took into account the quality of the theory of change. Was the ToC presented at a sufficiently disaggregated level to link activities undertaken at the grassroots with outcomes? Were key components of the ToC elaborated including contextual factors and assumptions?

Finally, agencies involved in L&A often have multiple sources of support, sometimes even multiple projects from the same donor. To what extent did the evaluation assess these additional sources of support as possible alternative explanations of the observed change? Was the contribution of the project being evaluated necessary?

We recognise that most of the reviewed evaluations had additional purposes, other than measuring the effectiveness of strategies. The key value of approaches like MSC and OH are that they use participatory methods of data collection, building ownership by stakeholders in the evaluation process. They uncover outcomes that the reviewers find most compelling, which can be seen as values inquiry (Renger & Bourdeau, 2004) or as the surfacing of potential criteria of merit.^4^ For this reason, the reader should note that our confidence assessments relate solely to confidence in the causal claims made and not the other purposes the evaluations may have had.

In order to avoid double-counting of any factors relating to confidence, 35 of the signalling questions were included in the calculations of overall and criteria-wise scores and assessments (indicated in Supplement 1). We applied a coding system: ‘yes’, ‘probably yes’, ‘probably no’, ‘no’ and ‘unclear’. We developed the assessment tool to avoid incentivising weak reporting on aspects where the conduct itself was weaker.^5^ The scores for relevant criteria were added together to reach an overall score for each evaluation.^6^ We used a decision algorithm to determine the final assessment, to ensure that those areas that we believed were more important in evaluating effectiveness did not receive the same weighting as less important ones.

The decision algorithm we used accorded with the criteria for confidence in an evaluation, outlined above. In order for the evaluation to be assessed as having ‘medium confidence’, the evaluation needed to define clearly the outcomes of interest (Q1.3), report effectiveness questions (Q1.6), posit plausible causal mechanisms linking activities to outcomes (Q2.5), adequately report sample characteristics (Q4.6) and use multiple, separate information sources (Q7.1). In order for the study to be assessed as having ‘high confidence’, the evaluation additionally needed to describe the capacity building and/or L&A activities that were undertaken (Q1.2), present a timeline showing that cause preceded effect (Q2.4), clearly describe the qualitative methodology used (Q2.7), present a theory of change with assumptions (Q3.1), present a stakeholder map (Q4.1), justify the sampling approach used (Q4.5), describe and justify the analysis process in sufficient detail (Q5.1, Q5.6), present an evaluation matrix linking evaluation questions to methods used (Q6.1), use appropriate sources of evidence (Q7.4) including those not involved in the programme (Q7.5), triangulate evidence (Q8.1), present alternative possible causal claims (Q9.1), attempt to rule out alternative explanations (Q9.2), attempt to protect against respondent bias (Q9.5) and evaluator bias (Q9.7), and clearly describe how data were collected from informants (Q10.3) and/or document review (Q10.4). All other evaluations were assessed as at ‘low confidence’, so such a study did not clearly report on the interventions or outcomes being measured, or define, conduct or report effectiveness questions, or report on the methods used to evaluate effectiveness.

Although we did not use a formal panel like a Delphi to develop the assessment tool, we aimed for a good process of stakeholder engagement in conducting this study, in order to be ‘empowering not extractive’ (Chambers, 2007) and therefore influential in the policy and evaluation communities. An external reference group (ERG), comprising three providers of qualitative impact evaluations and five users and commissioners of the evaluations assessed in this study, was brought together in the first month of the project and met four times during the study. We elaborated the approach to assessing the strength of evidence consultatively, including by sharing the preliminary assessment tool with the ERG for comments. In addition, the preliminary findings of the assessments and outcomes collected from each evaluation were shared with each programme organisation and evaluation partner, who were able to comment on and challenge the codes that had been assigned.^7^ We subsequently updated the preliminary coding for each study, as appropriate, based on the feedback. We held a public consultation on the findings with a large stakeholder group involving around 80 people from government, NGOs and the evaluation community in the Hague in December 2022, which was facilitated by IOB.

## Results

We assessed the methods used in, and collected the findings from, external end evaluations of effectiveness of 28 D&D^8^ and seven SRHR Partnership Fund programmes, providing over Euros 1.2 billion worldwide in 2016–2020 (Table 1).^9^ Examples of D&D programmes were Freedom from Fear (Euro 50 million), Global Alliance for Green and Gender Action (GAGGA) (Euro 32 million), Partnership to Inspire, Transform and Connect the HIV response (PITCH) (Euro 50m) and Towards a Worldwide Influencing Network (Euro 78 m). SRHR programmes included Bridging the Gaps (Euro 50 million) and More than Brides (Euro 59 million).

**Table 1.** Characteristics of Programmes Included in Meta-Evaluation.

Policy area	Programme Name	Lead Organisation	Countries	Budget (Euro Millions)
D&D	Freedom from fear	Pax	13	59.5
D&D	Fair green and global alliance	Both ENDS	36	59.5
D&D	Shared resources and joint solutions	IUCN	16	59.5
D&D	Towards a worldwide influencing network	Oxfam Novib	23	77.8
D&D	Building capacity for sector change	UTZ	9	18.3
D&D	PRIDE	COC	16	18.3
D&D	Garment supply chain transformation	Fair Wear Foundation	9	32.1
D&D	Health systems advocacy 4 Africa	Amref	5	32.1
D&D	Conducive environments for effective policy	NIMD	14	32.1
D&D	No news is bad news	Free Press Unlimited	21	32.1
D&D	Advocacy for change	Solidaridad	6	32.1
D&D	GAGGA	FCAM	31	32.1
D&D	Count Me in!	Mama Cash	31	32.1
D&D	Girls advocacy alliance	Plan Nederland	16	41.2
D&D	Green livelihoods alliance	Milieudefensie	9	41.2
D&D	PITCH	Aids Fonds	17	41.2
D&D	Partners for resilience (PfR)	Rode Kruis	10	43.1
D&D	Citizens agency Consortium	Hivos	20	50.4
D&D	Prevention up front	GPPAC	38	10.0
D&D	Watershed - empowering citizens	IRC	6	16.4
D&D	Every voice Counts	Care	6	16.4
D&D	SNV and IFPRI alliance	SNV	7	34.7
D&D	Empowering people in fragile contexts	Cordaid	9	34.7
D&D	Right here, right now	Rutgers	18	34.7
D&D	Civic engagement alliance	ICCO	14	34.7
SRHR	Bridging the gaps	Aids fonds	11	50.0
SRHR	Yes I do	Plan	5	27.6
SRHR	GUSO	Rutgers	7	39.6
SRHR	Jeune S3	Cordaid	4	29.8
SRHR	Her choice	Stichting Kinderpostzegels	10	18.7
SRHR	Down to zero	Terre des hommes	10	15.0
SRHR	More than brides	Save the Children	10	58.6
D&D	D&D programme total			916.3
SRHR	SRHR partnership fund total			239.3

The most frequently used methods to evaluate the programmes were outcome harvesting and, sometimes alongside, contribution analysis, or combined with another method such as MAPP, MSC or realist evaluation.^10^ But 17% of the evaluations used an unclear method to evaluate the effects of the programme. This is not equivalent to saying that they did not specify data collection approaches (like FGDs and document review) or sampling procedures, for example. They often did, but they did not describe an evaluation design or method used to ascertain effectiveness (i.e. the contribution of the programme to the outcomes achieved). Articulating the approach to collecting data is necessary but insufficient for determining how the effectiveness question is to be addressed.

Table 2 presents a summary of the results of the assessments, showing the average score and percentage of possible maximum score, together with the maximum and minimum scores for each criterion and overall, for D&D and SRHR programmes (complete scores for each evaluation are in Supplement 2 Tables 2.1 and 2.2). In general, the assessment indicated evaluations for both types of programmes which were assessed favourably (scoring more than 70%) on criterion #8 (triangulation of results using different information sources). However, the average score was low (below 30%) for methods to protect against biases (e.g. evaluator bias and respondent bias). Greater challenges were identified for D&D than SRHR, particularly with regard to choice of indicator (criterion #3), sample selection (#4), appropriate analysis (#5) and description of data collection and analysis (#10), as discussed here.

**Table 2.** Results of Assessments by Programme Type.

Criteria	Criteria Description	Max Possible Score	D&D				SRHR			
			Average Score		Max Score	Min Score	Average Score		Max Score	Min Score
#1	The research design is clearly elaborated and shows how the research results will contribute to answers to the evaluation questions	12	8.5	71%	12	5	7.5	63%	12	2
#2	The methods are appropriate to evaluate effectiveness: Attribution and/or contribution	12	5.3	44%	12	0	5.6	47%	9	3
#3	The indicators or result areas are appropriate to capture the planned results along the different levels in the ToC	12	3.6	30%	9	0	5.5	46%	8	3
#4	Justified choice of sample, cases and information sources (e.g. choice of countries, projects, organisations and persons)	9	2.7	30%	9	0	4.5	50%	7	2
#5	The analyses are appropriate, given the chosen research design	6	2.8	46%	6	0	4.5	75%	6	2
#6	Summary of the methodology in an evaluation matrix and criteria #17 sufficient independent information sources	3	1.4	45%	3	0	0.6	21%	3	0
#7	Sufficient independent information sources	12	5.7	47%	12	0	5.1	43%	10	3
#8	Triangulation of results from different information sources	3	2.1	71%	3	0	2.1	71%	3	0
#9	Discussion of bias	24	4.0	17%	12	0	3.4	14%	10	0
#10	Systematic, complete and transparent description of the data collection and analysis	6	2.0	33%	6	0	2.9	48%	4	0
#11	Discussion of the limitations of the evaluation	6	3.3	55%	6	0	5.0	83%	6	2
Total		105	41.3	39%	73	19	46.8	45%	56	37

Regarding research design (criterion #1), evaluation questions regarding effectiveness were usually clearly presented. The interventions were often clearly described (Q1.2a and 1.2b) as part of their achievement (that the planned activities were carried out). But for over half of the evaluations the programme timeline was not clearly stated showing that implementation of the intervention preceded the observed change in outcome. Frequently, OH was used because the programmes organisations had built outcome monitoring into internal monitoring, evaluation and learning (MEL) systems, providing a participatory approach to building capacity in results-based management. Drawing on these initial harvests, the evaluators would then conduct substantiation, where credible outcomes were triangulated with information from other sources. This was undertaken in some, but not all, causes. Sometimes, although more rarely, this approach was combined with another method like CA, MSC or MAPP. Regarding whether the evaluations were conducted appropriately, deviations from the standard method were tolerated where the evaluators needed to tailor it depending on the programme contexts and conditions (e.g. restrictions on travel imposed by the COVID-19 pandemic). However, the standards of reporting were often inadequate for our assessment, and this lack of clarity was also reflected in our assessments of the causal claims that were being made.

An important factor for identifying the causal relationship between what was done and what was achieved (temporal precedence) is that the changes should happen after the intervention (criterion #2). Few studies clearly described the timeline showing that implementation of the intervention preceded the observed change in outcome. There was often a lack of clarity on the programme components. These ‘missing beginnings’ were particularly noticeable at the country and grassroots levels. It was also not usually clear what the relative contribution of the programme was to the outcomes observed, or the causal pathways or mechanisms of change.

Rather than clearly describing causal mechanisms, many evaluations included statements like ‘the change was observed after implementation of the intervention’, followed by concrete examples of changes in outcomes. Although this sort of statement is suggestive of effectiveness, it is insufficient to confirm causality. The minimum requirement for articulating cause and effect is to specify what was done, for whom, when and with what observed outcome. Therefore, studies that presented plausible posited causal mechanism did so by articulating it through the ToC (i.e. specific inputs, activities, outputs and outcomes). Yet examination of alternative hypotheses was rarely done (Q1.6).

To take one representative example that combined OH with MSC, there was a missing step in the evaluation methodology between the MSC stories harvested and the presentation of findings in the report; in particular, there was no explicit verification of the MSC stories or discussion of how triangulation with interviews, FGDs and document review was used to determine which MSC stories were more or less credible, which the evaluators said was partly due to inability to travel during COVID-19. Similarly, while high level barriers and enablers to effectiveness were discussed, there was limited discussion of alternate explanations for the outcomes achieved at the grassroots level. And, relatedly, there were ‘missing beginnings’ at the grassroots level, so while the programme theory was clearly mapped using the global ToC and data presented on activities and outputs at the high level, there was very limited reporting of activities at the grassroots level to demonstrate temporal precedence – that the actions undertaken by CSOs was related to the actions of the programme itself – hence the issue arose about whether capacity was being built or utilised.

As noted in the background section above, a programme theory of change (criterion #3) was usually presented which incorporated a list of outcomes, but this was usually missing some crucial information, such as underlying assumptions, project participants and contextual and external factors (Q3.1). Measurable indicators linked to these outcomes were not always provided, especially for D&D programme evaluations, and other aspects of ToCs were missing, for example, lack of underlying assumptions and articulation of theoretical links. An example of a measurable indicator was the existence of inclusive policy and law-making processes measured under the outcome ‘inclusion of voice of [female] smallholders’. The ToC could have been presented as an ex-ante programme theory or as ToCs for specific interventions or activities that were eventually undertaken.

Outcomes were measured in the areas of capacity development, support to lobby and advocacy efforts, policy engagement, policy change, empowerment and access to SRHR services (*n* = 1143). For D&D programmes, the most frequently measured outcomes were policy engagement (*n* = 198; 21%), policy change (*n* = 110; 11%) and policy implementation (*n* = 161; 17%). In contrast, capacity development of CSO partners was measured relatively infrequently (*n* = 59; 6%), and when it was done the measurement was not of the value added to CSO L&A capacity in comparison with existing knowledge, attitudes and/or practices. Potential unintended outcomes (e.g. spillover effects) were not presented in ToCs, while in some evaluations unintended outcomes, including negative effects that were observed were reported in the findings sections without linking them to the programme ToC (Q3.4).

The sample characteristics for interviewees were often adequately reported (criterion #4). The lists of interviewees and documents reviewed were often presented (Q4.2 and 4.3). Accordingly, the sample characteristics for interviewees were often adequately reported (Q4.6). However, stakeholder maps were only given (Q4.1) in one case of SRHR and two D&D programmes, leading to the potential for omitted informant bias. Weaknesses in the sampling strategy were noted where the evaluation used OH and only the selection of countries was justified, and the selection process of interview participants was not clearly described. Sample selection processes were not adequately described and rarely justified for D&D programme evaluations (Q4.4 and 4.5). For example, one report presented only how country case studies were selected, but not how interview participants were recruited. As a result, it was not possible to judge appropriateness of the sampling strategy in more than half of the evaluations (Q4.7).

The analysis process (criterion #5) was mostly described in detail for SRHR programme evaluations. However, in more than half of the D&D evaluations, which concrete steps were taken, and therefore whether the evaluation conduct was appropriate, was unclear (Q5.2–5.5). For example, in the case of OH, the criteria for outcomes that were reported were often not clearly described. While, in general, the description of data collection was clearly presented, the data analysis process was not clearly given (Q5.6).

An evaluation matrix (criterion #6) should clearly articulate how the evaluation questions are linked to the methods and approaches taken to data collection and analysis. Most studies presented an evaluation matrix (Q6.1) including evaluation questions and data sources, but few included the data analysis approach linked to each evaluation question.

Regarding sources of information (criterion #7), the evaluations generally used different types of information sources such as documents, interviews, workshops and focus group discussions, to triangulate the outcomes observed (Q7.1). The list of interviewees often suggested that appropriate sources internal to the programme were sought (Q7.4). Yet more than half of the D&D programme evaluations did not attempt to guard against subjective selection of cases (Q7.3) as they did not include information about respondent selection. Few evaluations, of either D&D or SRHR programmes, clearly indicated that relevant sources external to the intervention, such as non-participants, organisations who may have experienced another intervention or those not targeted by interventions like trade unionists, were consulted (Q7.5).

Recruitment problems were rarely discussed, so it was also not clear whether (and why, if any) some people selected for sample collection chose not to participate (Q7.6). For SRHR programme evaluations, the data collection more clearly attempted to mitigate cherry picking of cases, either through random sampling or through purposive selection from diverse groups to reflect different points of view. Half of the SRHR evaluations discussed recruitment issues; one study, for example, mentioned security issues in the programme countries as a reason why data could not be collected from some areas (Q7.6). Assessing the appropriateness of data sources was often impossible, as relevant information was not found.

In most cases, triangulation (criterion #8) was incorporated as an essential part of the evaluation design. This was usually done through data triangulation, using data from different locations, times and participants. For example, for the method most commonly adopted, OH, outcomes harvested by programmes teams were subject to validation or substantiation through further interviews by the evaluators. But ‘methodological triangulation’ was also done in some cases, for example, by combining two evaluation approaches (e.g. OH with CA or quasi-experimental design with qualitative data on processes). Investigator triangulation, where multiple investigators compared notes after interviews, was only reported in two evaluations, which used more than one interviewer to collect the data or provided space for different perspectives among the evaluation team.

However, few evaluations attempted to rule out important sources of bias that affect any causal study (criterion #9), namely, alternative causal claims (Q9.1), contributory factors (Q9.2) and predictable respondent or evaluator biases. While data triangulation was often used as a way to mitigate against bias, it is unlikely that data triangulation can address all sources of bias.

Two evaluations presented implementation issues and contextual factors that might have affected the outcomes. For example, one evaluation linked policy engagement outcome achievements with effective L&A strategies implemented under the D&D programme, as well as external enabling factors, including decentralisation in the energy sector which provided opportunities to provide local technical support, and general concerns in the international community and general public about climate change and enabled agenda setting by the CSO partners (Sloot et al., 2020). The evaluation also linked desired policy changes around harmful fossil fuel subsidies, which were not achieved, to external political and civil changes, the discovery of fossil fuels that Dutch embassies supported, and internal factors such as the collaborative advocacy approach that was adopted which made it harder to criticise fossil fuel companies openly. Apart from this, no clear statements on alternative causal explanations were found. One evaluation team stated that they explored the potential contribution of other actors, using the principles of contribution analysis, but did not discuss these actors. As a result, we observed that the attempt to rule out competing causal explanations or contributory factors was not done in most of the evaluations using small-n approaches.

Potential biases caused by evaluators’ own positions and assumptions (Q9.4) were rarely mentioned. One type we thought might be discussed related to confirmation bias, which can be mitigated, for example, by recording interviews and comparing notes by multiple interviewers (Q9.7). Only a few studies suggested that this sort of mitigation measure was taken. One evaluation indicated coders were blinded to coding completed earlier by the organisational MEL during an outcome mapping session. Two others attempted to mitigate evaluator bias through investigator triangulation, although less clarity was given on whether and how the investigators’ notes were compared.

Regarding respondent bias (Q9.5), forms of social desirability bias such as courtesy bias, errors of attribution (positional bias), and errors of positioning oneself or one’s organisation at the centre of events (self-importance or self-serving bias) can be mitigated by, for instance, drawing up interview schedules to avoid leading questions, or blinding participants to the purpose of the evaluation (i.e. not mentioning the intervention, at least early in interviews). Only a few studies mentioned this bias. Interview protocols often began with questions about the programme, before asking about achievements and possible causal claims, which is a clear form of anchoring bias. To mitigate respondent bias, one evaluation incorporated ‘Social Presencing Theatres’ in focus group discussions. Another used what they called a ‘double blind’ methodology, meaning that neither the researcher nor interviewees knew who the client was, although it should be noted this is not the same as masking of knowledge about participation in the intervention. One-third of the evaluations attempted to protect against recall bias (Q9.6). For example, OH was started in the final year of implementation in one programme evaluation. But while it was usually clear when the evaluation was conducted, in many cases it was unclear when the data were collected with respect to the interventions and outcomes.

The processes for collecting data (criterion #10) were clearly presented in most of the evaluations, indicating how, when and with whom interviews, workshops and FGDs were conducted and recorded. Often, questionnaires and/or interview protocols were presented (Q10.3). Less clear were whether data codes, categories or themes were structured around the ToC (Q10.1). Regardless of the evaluation methodology, results and findings sections and analysis protocols were rarely clearly linked to the ToCs – although where OH was used, it can be inferred that each outcome category observed was referred to the ToC by the nature of the methodology. Few evaluations linked their data collection protocols to possible alternative hypotheses (Q10.2). Document review was conducted in most evaluations, and the list of documents given, but it was unclear what data collection codes were used (Q10.4).

Most reports included a summary section that clarified the link between the findings and evaluation questions (11.1). Similarly, the implications or recommendations were clearly linked to the findings, by virtue of this section usually coming immediately afterwards, hence the link was clear (11.3). Almost all of the evaluations discussed limitations (11.2) where the evaluators explored various challenges from risk of bias to limited data availability due to the pandemic, with different depths of exploration. Limitations due to data or resource availability as a result of the COVID-19 pandemic were usually mentioned. Some evaluations attempted to mitigate the resulting biases. Most SRHR evaluations clarified that their research complied with ethics (anonymity, informed consent and confidentiality). But, for nearly 20 D&D programme evaluations, we could not confirm that the research complied with ethical standards (anonymity, informed consent and confidentiality). There is a stronger tradition in evaluation in the health sector to address ethical issues regarding the collection and use of personal information. Feedback on the preliminary assessment from the NGOs and evaluators revealed that ethical issues were considered, but not reported.

The overall score for D&D programmes was lower at 39 percent, compared to 45 percent for SRHR programmes (Table 2). These findings, and intuition, suggest that it is simply more difficult to design studies to evaluate the effectiveness D&D credibly. Unlike SRHR, however, two D&D programme evaluations were assessed as having ‘high confidence’ overall in the findings. Six evaluations of SRHR programmes and 13 of D&D programmes were assessed as being of ‘medium confidence’ (Table 3). The remaining studies were assessed at ‘low confidence’ in the findings regarding effectiveness.

**Table 3.** Confidence Ratings by Programme Type.

Rating	Description	D&D			SRHR		
		Low	Medium	High	Low	Medium	High
Overall confidence	The assessment of confidence in the findings about effectiveness	13 (46%)	13 (46%)	2 (7%)	2 (25%)	6 (75%)	0

The majority of the outcomes measured in the evaluations were reported to be positive changes. Regarding confidence in the outcomes harvested, the evaluations rarely provided a clear explanation of the contribution of the programme activities to the outcome and the strength of the evidence, and where these were reported, they were often rated as ‘medium’ or ‘strong’. Hence, in 69% of the outcomes harvested for D&D and 82% for SRHR, the reported contribution was unclear (Table 4).

**Table 4.** Summary of Outcomes Reported in the Evaluations.

	D&D	SRHR
Positive outcomes	89	80
Null (no change) outcomes	9	11
Negative outcomes	2	8
Reported contribution	Unclear – 69; medium or strong – 30	Unclear – 82; medium or strong – 17
Reported evidence rating	Unclear – 66; medium or strong – 33	Unclear – 82; medium or strong – 18

*Note.* Figures are percentages, which may not sum to 100% due to rounding errors.

## Discussion

In order to address a question about effectiveness, meaning attribution or contribution of a programme to the defined outcome(s), it should be clear when and for whom the intervention was undertaken, what outcomes were achieved, and what were the likely causal pathways (intermediate outcomes) and contextual factors that might provide competing possible explanations or contributory factors. This implies, firstly, that evaluations should be based on, and reported around, a programme theory of change. ToC is a crucial step of many qualitative approaches, and is important more generally in programme evaluation. For example, a clear advantage of articulating the ToC is to avoid the problem of ‘premature impact evaluations’, where data are collected and analysed before changes in outcomes can be realised. We note that ToCs detailing all of the activities and strategies pursued are not necessarily generated ex-ante by programmers, and may not be desirable in adaptive programmes anyway. However, in these instances, presentation of (ex-post) ToC(s) by evaluators can provide useful information about the causal pathway, and where it might break down. Secondly, it implies that some method is needed to substantiate causal claims being made and to articulate the likely contribution of the programme activities to the outputs and outcomes achieved. These conditions are most likely to be met for theory-based methods such as contribution analysis and process tracing.

We also reiterate that the purpose of the evaluations reviewed may not have been primarily about confident attribution or contribution, but rather to answer other questions about implementation. Indeed, most of the evaluations reviewed did have multiple purposes. Hence, our findings with respect to confidence relate solely to the effectiveness question and not to the other questions posed. We believe that a review of causal claims is worthwhile even though a mix of purposes was present across the evaluations. But it is important for readers to understand that the standards we have developed and applied in the meta-evaluation may not always fully match the motivations behind the evaluations.

However, the application of appropriate qualitative causal inference approaches can clearly be strengthened. This meta-evaluation found that few evaluations properly applied a qualitative causal inference approach that systematically unpacked and assessed causal mechanisms, and resulted in substantiated causal pathways vested in an explanation of how an intervention is leading to a change in a particular context (i.e. taking into consideration other causal factors). Gardner and Brindis (2017) argue for greater use of contribution analysis and similar approaches in advocacy and policy change evaluation to analyse systems change, together with experimental and quasi-experimental approaches to assess changes in quality-of-life outcomes among target populations, but note the latter may not be appropriate methods to link to advocacy efforts themselves.

Regarding data collection we note that, for some outcomes, the measure was relatively strong, in that it was objectively verifiable (e.g. a policy change), but the contribution story less credible owing to the length of the causal pathway. In other cases, where the causal pathway was short, the contribution story to immediate outcomes (e.g. capacity building) was potentially strong but the measures of change were often weak. For immediate outcomes relating to capacity building, it would be useful to incorporate, as a validation method, more objectives assessments of knowledge and practices of CSO staff before and after the programme was implemented. It is important to assess CSO capacity in order to evaluate whether competent L&A was working, even if it had no effect on endpoint outcomes like policy change and implementation; for example, this might include the capacity to keep campaign material ready until the time is right.

For credible assessments of the effectiveness of programmes in achieving outcomes further along the causal pathway, evaluators and evaluation commissioners should ensure that both successes and failures in achieving outcomes, and alternative explanations, or contributions, by external actors to the achievements of programmes, are measured. We acknowledge that contextual factors such as shrinking civic space influence the options of feasible evaluation methods. However, evaluations that draw closely from the programme ToC should also be able to engage better with outcomes that were not achieved, and collect data to help understand why that was the case. It is also important that the outcomes harvested by programmes staff and/or participants are, firstly, substantiated by evaluators through enquiry and data triangulation. Secondly, there is likely to be a need for additional analysis by the evaluators to assess the possibility of alternative causal pathways, in order to address contribution, or to assess outcomes that were part of the programme theory of change, but did not occur. The latter might be achieved through enquiry and analysis of outcomes that were part of (pre-specified by) the programme theory, but not necessarily mentioned in outcomes harvested by programmes staff.

There is also the issue of probative value when selecting informants, where the choice of respondent is related to the quality of the information provided since not all data sources are of equal value (Vaessen et al., 2020). It was rarely clear how the choice was made about whom to sample among those participating in the programmes (usually grassroots CSO and programmes staff) and those that did not participate who might have had different, but informed, perspectives about the programme’s achievements. Where projects work in multiple global regions and countries, with multiple partners, a suitable approach is to provide a descriptive overview of the portfolio at global or regional level, and evaluate effectiveness in a small sample of cases (countries, projects or L&A trajectories) chosen according to some transparent selection process. This approach was adopted by many of the programme evaluations, although the justification for the cases chosen was usually unclear.

Regarding our confidence in the outcomes achieved, the evaluations rarely provided a clear explanation of the contribution of the programme activities to the outcome and the strength of the evidence, and where these were reported, they were often rated as ‘medium’ or ‘strong’. Hence, the contribution of the L&A support to the outcomes reported was often unclear.

There also appeared a clear bias towards reporting positive effects. This is likely in part due to low incentives to delve into negative effects or elucidate null effects; ‘self-serving bias’ or ‘intervention-centric bias’ leading to an overestimation of the role of an intervention vis-à-vis other causal factors; and methods choice. Some methods (e.g. OH and MSC) may enhance a bias towards positive effects reporting, requiring work on the part of the evaluators to explicitly capture alternative causal pathways or to assess outcomes that were intended but did not occur.

Drawing on the evaluations that were assessed as being at ‘low confidence’, we present a synthetic example of an approach that would not in our view be able to address contribution or attribution of programmatic support to L&A:

• The evaluation did not collect data on outcomes that were intended in the theory of change but not achieved, which would help to understand what didn’t work, as well as what did.
• The evaluation did not specify a method that was used to assess causal claims. For example, where a case study approach was used to collect and analyse data, it did not articulate relevant causal pathways using the ToC.
• Where the approach did specify a method used to evaluate effectiveness, the outcomes collected were not independently verified by the evaluators through data triangulation.
• Outcomes were collected from those who were part of the programme or who participated in the L&A activities, but not from informed outsiders that did not participate.
• There were ‘missing beginnings’, so there was very limited analysis of activities, especially at the grassroots level, to demonstrate that the L&A actions undertaken by CSOs were related to the actions of the programme itself.
• There were weak measures of outcomes, such as on capacity building, or, where outcome measures were potentially strong, ‘missing middles’ that demonstrated feasible causal pathways to their achievements from the activities undertaken.
• Predictable biases were not avoided. For example, interviews commenced with questions about the programme of interest and proceeding to ask about possible outcomes, a clear example of anchoring bias.
• No attempts were made to analyse alternative causal pathways that may have contributed to the outcomes being achieved (or not), whether other interventions that were occurring at the same time or contextual factors.

We believe the findings from the meta-analysis are relevant for evaluations of aid effectiveness in lobby and advocacy and small-n qualitative impact evaluation more generally. We assessed 36 evaluations of multi-component international programmes to support civil society L&A across Asia, Africa and Latin America, comprising the majority of programmatic support from one international donor. We reviewed the literature on evaluating L&A and small-n impact evaluation, finding no assessment tool to assist users of the evaluations in placing confidence in the findings from the evaluations. The approaches we advocate are useful in fostering the role of evaluation in assessments for both accountability and learning (Kogen, 2018; Picciotto, 2018).

The main limitation of this study is that we were restricted to a desk review of the available technical reports and annexes that were provided to us. As discussed above, we obtained detailed feedback from the programmes organisations and evaluators on the initial coding, which was updated accordingly, as appropriate, and therefore served as an additional quality check on the coding undertaken. In some cases, the organisations provided additional information relating to the evaluation design (e.g. inception reports), which we were able to incorporate in the assessments. Hence, we believe the final assessments to be an accurate reflection of the confidence an informed user would place in the findings of the evaluations.

## Conclusions and Implications for Evaluation Practice

We identified areas where evaluation design, conduct and reporting can be improved in small-n qualitative impact evaluations. These included: providing more information about interventions occurring at the country and grassroots levels, to avoid the problem of ‘missing beginnings’ in the theory of change; justifying the choice of sample and avoiding ‘omitted informant bias’ from those who may not have participated in the programme but who might have had different, but informed, perspectives about its achievements; and addressing predictable sources of bias in establishing effectiveness relating to alternative factors contributing to change, and respondent and evaluator biases. The reporting of causal claims could also have been clearer in many cases, by explicitly using the theory of change to link programme inputs and activities with outputs and outcomes, especially at the country and grassroots levels, to avoid the problems of ‘missing beginnings’ and ‘missing middles’, and to ensure that the full range of possible outcomes were evaluated, not just those that resulted from effective strategies.

While appropriate evaluation methods can vary from one programme to another, depending on its context, budgetary constraints, programme size and sample size, further guidance could be provided to evaluators on the methods that can be used to evaluate causal claims for particular types of programmes and outcomes. Some studies clearly stated that their evaluations focused on contribution, but others did not, which suggested that, for evaluations of L&A effectiveness, guidance would be especially helpful for devising evaluation questions that more clearly address contribution or attribution.

Research may be needed to assess whether it is possible to address these predictable biases that arise in the data collection and analysis process of methods commonly used to evaluate L&A, such as outcome harvesting. For example, collecting data from a sub-sample of non-participating organisations or groups that were part of or influenced by other programmes, may also help in identifying drivers of change external to the programme being evaluated in OH. Or blinding of investigators might be possible, but even in a non-blinded evaluation, anchoring bias can and should be avoided through careful interview design.^11^ Research is also needed on appropriate methods that are suitable for evaluating effectiveness in the lobby and advocacy for systemic changes (e.g. public and private sector actors’ policies and practices).

Evaluations of L&A programmes – and qualitative impact evaluation more generally – can also be improved through clearer guidance on the design and conduct of evaluations, ensuring they engage with outcomes that are achieved and those that are not. Guidelines and checklists are commonly provided in other areas of evaluation (e.g. Moher et al., 2010; von Elm et al., 2007; Page et al., 2021; White et al., 2020). The distinction between issues that relate to design and conduct, and those which relate to reporting, are important. We suspect that, in the evaluations we reviewed, some low scores were likely to be due to reporting failures rather than conduct failures in evaluations (e.g. investigator triangulation may have been implemented in a number of cases but was not mentioned in the methodological sections or appendices of the report).

Other approaches, used in other areas of monitoring, evaluation and learning, include specific efforts by commissioners to ensure that there is learning from intended outcomes that were not achieved, such as celebrating failure (e.g. ‘failure fests’). At a minimum, CSO and government commissioners should indicate that evaluations clearly draw on a credible theory of change, and aim to collect data on all relevant outcomes, not just those that were successfully achieved, in order to learn from successes and failures more systematically.

## Supplemental Material

Supplemental Material - What can we Learn From Qualitative Impact Evaluations About the Effectiveness of Lobby and Advocacy? A Meta-Evaluation of Dutch aid Programmes Supplemental Material for What can we Learn From Qualitative Impact Evaluations About the Effectiveness of Lobby and Advocacy? A Meta-Evaluation of Dutch aid Programmes by Hugh Sharma Waddington, Hikari Umezawa and Howard White in Evaluation Review

## Notes

1. An in-depth assessment and synthesis of the outcomes harvested is contained in Sharma Waddington et al. (2023).

2. For a survey, see White and Phillips (2012); for methods guidance, see Vaessen et al. (2020).

3. The literature was identified by Google Scholar and Google searches.

4. We thank the anonymous referee for this suggestion.

5. We note that ‘unclear’ meant that there was insufficient reporting of information in order to address the signalling question. This lack of clarity therefore primarily related to lack of information about what was done. While this may have also led to a lack of clarity in our own judgement, the primary evidence on which the assessment is based relates to transparency around conduct and reporting.

6. The following scoring system was applied: Yes = 3, probably yes = 2, probably no = 1, no = 0 and unclear = 0.

7. Feedback on the preliminary coding revealed that many low scores came about through inadequate reporting rather than inappropriate conduct.

8. The original remit was to evaluate 25 D&D programmes and 7 SRHR Partnership Fund. However, one programme from D&D programme, the Citizen Agency Consortium, contained four separate evaluations which were assessed individually, bringing the total to 28; these were Sustainable Diets for All, Green and Inclusive Energy, Decent Work for Women, and Open Up Contracting. One programme from SRHR Partnership Fund (the More than Brides Alliance) contained two evaluations (one on Pakistan, the other on India, Malawi, Mali and Niger), resulting in eight evaluation reports to be reviewed.

9. The evaluations were commissioned to address the Dutch Government’s Evaluation Quality Criteria that existed at the time the evaluations were designed. Therefore, the assessments are not performance reviews of those conducting those evaluations. For this reason, we have anonymised the discussion of the assessments.

10. Three evaluations of SRHR programmes also incorporated quasi-experimental design; that is, they compared results for participants who received the SRHR intervention with a comparison group which did not.

11. For example, the Qualitative Impact Protocol (QuIP) has overlapping features with qualitative methods like outcome harvesting but addresses the attribution challenge explicitly by attempting to eliminate bias by focussing data collection on all drivers of change for the outcomes of interest with both respondents and enumerators who are ‘blindfolded’ to the intervention being evaluated, thus reducing respondent and evaluator biases and potentially addressing attribution (Copestake et al., 2019). This approach is evidently possible in evaluations of L&A, especially where they are conducted by consortia that incorporate or are led by local partners.
