# Supplementary material for: What Can We Learn From Qualitative Impact Evaluations About the Effectiveness of Lobby and Advocacy? A Meta-Evaluation of Dutch aid Programmes and Assessment Tool
Source: Eval Rev. 2025 Jan 11;49(5):851–79. doi: 10.1177/0193841X251314731 (PMC12379041; doi:10.1177/0193841X251314731)
Supplement: Supplemental Material - What can we Learn From Qualitative Impact Evaluations About the Effectiveness of Lobby and Advocacy? A Meta-Evaluation of Dutch aid Programmes [file sj-pdf-1-erx-10.1177_0193841X251314731.pdf]

Title: What can we learn from qualitative impact evaluations about the effectiveness of lobby and advocacy? A meta-evaluation of Dutch aid programmes

Supplementary materials

Supplement 1 Coding form

#	*Signalling question*	*Notes on signalling question*	*Responses*	*Included in score?*
**#1 The research design is clearly elaborated and shows how the research results will contribute to answers to the evaluation questions**				
1.1	Are the interventions of interest named or identified?		Y/PY/PN/N/UC/NA	
1.2a	Are the capacity building interventions clearly described, including implementation timelines?		Y/PY/PN/N/UC/NA	Yes
1.2b	Are the L&A interventions clearly described, including implementation timelines?		Y/PY/PN/N/UC/NA	Yes
1.2c	SRHR only: Are the service delivery interventions clearly described, including implementation timelines?		Y/PY/PN/N/UC/NA	Yes
1.3	Are the outcomes of interest clearly defined? List all outcomes with definitions.		Y/PY/PN/N/UC/NA	Yes
1.4	Is the intervention context described adequately, including contextual/external factors, such as social/cultural setting, political or economic factors, and parallel interventions or other stakeholder actions?		Y/PY/PN/N/UC/NA	
1.5	Are programme participants and project-affected persons clearly described?		Y/PY/PN/N/UC/NA	
1.6	Are the evaluation questions regarding effectiveness clearly stated?		Y/PY/PN/N/UC/NA	Yes
1.7a	What approaches do the evaluators say they planned to use to measure attribution or contribution of capacity building intervention(s) to outcome(s)?		Open-ended question	
1.7b	What approaches do the evaluators say they planned to use to measure attribution or contribution of L&A intervention(s) to outcome(s)?		Open-ended question	
1.7c	What approaches do the evaluators say they planned to use to measure attribution or contribution of service delivery intervention(s) to outcome(s)?		Open-ended question	

#	*Signalling question*	*Notes on signalling question*	*Responses*	*Included in score?*
1.8	What approaches have they actually used to assess attribution?	*Using 'large n' approaches, by measuring outcomes with respect to a comparison group, using a method like difference-in-differences*	Open-ended question	
1.9	What approaches have they actually used to measure contribution?	*Using 'small n' approaches like Realist Evaluation, GEM, Process Tracing, Contribution Analysis, MSC, SCM, Outcome Mapping, MAPP, or something else?*	Open-ended question	
1.10	Does the approach belong to Group 1 (more explicit causal identification) or Group2 (more participatory approach), as defined by White & Phillips (2012)	*Group 1: realist evaluation, general elimination methodology, process tracing, contribution analysis*<br>*Group 2: most significant change, success case method, outcome mapping, outcome harvesting, MAPP*	Y/PY/PN/N/UC/NA	
**#2 The methods are appropriate to evaluate effectiveness: attribution and / or contribution**				
2.1	Is the causal claim clearly stated?	*For example, "XXX caused/led to/ contributed to/impacted/affected YYY"… "Without XXX, YYY might not have happened…"/ "Otherwise, YYY would have not been possible... "*	Y/PY/PN/N/UC/NA	
2.2	Is the effect on the outcomes observed and reported?		Y/PY/PN/N/UC/NA	
2.3	Is a change in outcomes observed relative to a comparison group (that is, a group that does not receive the intervention of interest)?		Y/PY/PN/N/UC/NA	
2.4	Is there a timeline showing that the cause (implementation of the intervention) preceded the event (observed change in outcome)?		Y/PY/PN/N/UC/NA	Yes
2.5	Is there a plausible posited causal mechanism underlying the relationship between intervention and outcome?		Y/PY/PN/N/UC/NA	Yes
2.6	Does the evaluation articulate alternative causal hypotheses, including the role of contextual/external factors, such as social/cultural setting, political or economic trends, and parallel interventions or other stakeholder actions, that may influence outcomes?		Y/PY/PN/N/UC/NA	Yes
2.7	Is the qualitative methodology, which will interrogate the relationship between intervention and outcome, described?		Y/PY/PN/N/UC/NA	Yes

#	*Signalling question*	*Notes on signalling question*	*Responses*	*Included in score?*
**#3 The indicators or result areas are appropriate to capture the planned results along the different levels in the ToC**				
3.1	Is the ToC presented for the intervention(s) being evaluated, that<br>- sets out underlying intervention logic and theoretical links<br>- outlines inputs, activities, outputs, intermediate and final intended outcomes<br>- lists programme participants and project-affected persons, timelines and indicators to monitor change<br>- provides assumptions and risks at each link in the chain<br>- provides contextual factors and external influences in causal chain?	*Y if all the conditions are met; Probably Y if 3-4 met; Probably No if 1-2 met; No if none of them are met.*	Y/PY/PN/N/UC/NA	Yes
3.2	Are measurable indicators presented for the intervention(s) being evaluated (Impact-Outcome-Output-Activities-Inputs), for example in a log frame?		Y/PY/PN/N/UC/NA	Yes
3.4	Does the ToC/log frame articulate possible unintended outcomes (e.g., spillovers)?		Y/PY/PN/N/UC/NA	Yes
3.5	Is the selection of outcome collected/changes observed justified with reference to the ToC or otherwise?		Y/PY/PN/N/UC/NA	Yes
3.6	What measures or measurement instruments or approaches are used to measure capacity of CSOs?		Open-ended question	
**#4 The choice of sample, cases and information sources is justified**				
4.1	Is a stakeholder map presented ('omitted informant bias')?		Y/PY/PN/N/UC/NA	Yes
4.2	Is the list of interviewees presented (e.g., in an appendix)?		Y/PY/PN/N/UC/NA	
4.3	Is the list of documents presented (e.g., in an appendix)?		Y/PY/PN/N/UC/NA	
4.4	Does the recruitment or sampling strategy describe how have the participants been selected?		Y/PY/PN/N/UC/NA	
4.5	Is the sample selection process explained and justified?		Y/PY/PN/N/UC/NA	Yes
4.6	Are sample characteristics adequately reported (sample size, location, and at least one additional characteristic)?		Y/PY/PN/N/UC/NA	Yes

#	*Signalling question*	*Notes on signalling question*	*Responses*	*Included in score?*
4.7	Is the recruitment or sampling strategy appropriate, including explaining why the participants selected were the most appropriate to provide access to the knowledge sought to answer the evaluation questions?		Y/PY/PN/N/UC/NA	
**#5 The analyses are appropriate, given the chosen research design**				
5.1	Is there a detailed description of the analysis process?		Y/PY/PN/N/UC/NA	Yes
5.2	Contribution analysis: according to what is reported, is the method implemented appropriately?	*Contribution analysis involves: 1) articulating ToC; 2) evaluating whether intervention activities implemented as set out; 3) chain of expected results (outcomes) shown as having occurred; 4) other influencing factors ruled out or relative contribution recognised.*	Y/PY/PN/N/UC/NA	
5.3	Outcome mapping: according to what is reported, is the method implemented appropriately?	*Outcome mapping involves: 1) articulating ToC "intentional design" and "boundary partners"; 2) collection of outcome, strategy and performance journals, which may incorporate Most Significant Change (MSC) analysis; 3) "evaluation planning" (data collection and verification)*	Y/PY/PN/N/UC/NA	
5.4	Most significant change: according to what is reported, is the method implemented appropriately?	*Most Significant Change (MSC) involves: 1) defining domains of change and timeframe; 2) systematic collection of stories from participants about (positive and negative) changes that occurred in their lives in the recent past, enquiries about why the changes occurred and were significant; 3) systematic review of stories of change by stakeholder panels; 4) verification of stories through additional data collection and possible quantification of changes; 5) comparison of most significant change stories with expected changes in ToC/log-frame*	Y/PY/PN/N/UC/NA	
5.5	Outcome harvesting: according to what is reported, is the method implemented appropriately?	*Outcome harvesting involves: 1) gathering data on potential outcomes to which change agent may affect and contributions by change agent; 2) verification through informant review of draft outcomes, usually in workshop, and evaluator assessment of plausibility and*	Y/PY/PN/N/UC/NA	

#	*Signalling question*	*Notes on signalling question*	*Responses*	*Included in score?*
		coherence; 3) substantiation of outcomes and contributions through additional data interviews; 4) categorisation and interpretation of outcomes		
5.6	Is the data analysis approach presented in sufficient detail and justified?		Y/PY/PN/N/UC/NA	Yes
**#6 Summary of the methodology in an evaluation matrix**				
6.1	Does the study present an evaluation matrix or plan linking evaluation questions with nature and sources of data, protocols for qualitative field work and categories for data analysis?		Y/PY/PN/N/UC/NA	Yes
**#7 Sufficient independent information sources**				
7.1	Are separate types of information sources used e.g., documents, interviews, focus groups, field visits?		Y/PY/PN/N/UC/NA	Yes
7.2	What are these separate sources of information (Government statistics, surveys conducted my other entities, etc.)?		Open-ended question	
7.3	Does the data collection attempt to guard against cherry picking of cases, such as through random sampling of targeted programme participants or purposive sampling across a diverse group using a sampling frame (e.g., including those who may have dropped out), or indicate methods taken to avoid convenience sampling of respondents?		Y/PY/PN/N/UC/NA	
7.4	Are appropriate sources included that were involved in delivering or receiving the intervention - e.g., participants, implementers, programme managers?		Y/PY/PN/N/UC/NA	Yes
7.5	Are relevant sources included that were not involved in, or may have experienced another, intervention - e.g., trade union members?		Y/PY/PN/N/UC/NA	Yes
7.6	Is there discussion of issues around recruitment (e.g., why some people chose not to take part)?		Y/PY/PN/N/UC/NA	Yes
**#8 Triangulation of results from different information sources**				

#	*Signalling question*	*Notes on signalling question*	*Responses*	*Included in score?*
8.1	Is the evidence of a causal relationship triangulated?		Y/PY/PN/N/UC/NA	Yes
8.2	Describe the method(s) of triangulation used	*-Data triangulation (location, time and participants)*<br>*-Investigator triangulation*<br>*-Theory triangulation (several theories)*<br>*-Methodological triangulation*	Open-ended question	
8.3	Are these methods appropriate to answer evaluation questions?		Y/PY/PN/N/UC/NA	
**#9 Discussion of bias**				
9.1	Are possible alternative causal chains/claims presented?		Y/PY/PN/N/UC/NA	Yes
9.2	Does the study attempt to rule out alternative explanations for changes in outcomes, such as analysis of alternative hypotheses or falsification methods (irrelevant interventions or outcomes)?		Y/PY/PN/N/UC/NA	Yes
9.3	Is the evaluator's own position, assumptions and possible biases discussed, in order to protect against evaluator bias (e.g., 'friendship'/'contract renewal bias')?		Y/PY/PN/N/UC/NA	Yes
9.4	Is the evaluator affiliation financially independent from the organization being evaluated?		Y/PY/PN/N/UC/NA	Yes
9.5	Does the study attempt to protect against respondent bias*?	*e.g.,*<br>*- by drawing up questions to avoid leading questions in interviews*<br>*- BLINDING participants to the evaluation*<br>** respondent bias includes: includes courtesy bias/ political correctness bias, positional bias (e.g. errors of attribution to intervention), self-serving bias, self-importance bias*	Y/PY/PN/N/UC/NA	Yes
9.6	Are the data collected within a sufficiently short time period from implementation of the intervention to protect against recall bias (e.g., interviews conducted while the programme is ongoing)?		Y/PY/PN/N/UC/NA	Yes
9.7	Does the study attempt to protect against evaluator bias by recording interviews and comparison of notes by multiple interviewers (confirmation bias)?		Y/PY/PN/N/UC/NA	Yes
9.8	Was the potential for conflict of		Y/PY/PN/N/UC/NA	Yes

#	Signalling question	Notes on signalling question	Responses	Included in score?
	interest considered and addressed?			
**#10 Systematic, complete and transparent description of the data collection and analysis**				
10.1	For factual information: are initial themes, categories and data codes structured around ToC/log-frame/results framework?		Y/PY/PN/N/UC/NA	
10.2	For counterfactual information: are data collection protocols linked to comparison groups or possible alternative hypotheses?		Y/PY/PN/N/UC/NA	
10.3	Is it clear how the data were collected from informants; e.g. is there a discussion of how interviews/FGDs were conducted and recorded?		Y/PY/PN/N/UC/NA	Yes
10.4	Is it clear how document reviews were conducted; e.g. is a data collection sheet containing codes presented?		Y/PY/PN/N/UC/NA	Yes
**#11 Discussion of findings and limitations of the evaluation**				
11.1	Do the findings address the evaluation questions?		Y/PY/PN/N/UC/NA	
11.2	Are all potential limitations thoroughly discussed (limitation due to data availability, resource (time/funds) constraints, risk of bias and any other sorts of limitation)?		Y/PY/PN/N/UC/NA	Yes
11.3	Are the implications or recommendations clearly linked to/based on the evidence from the study?		Y/PY/PN/N/UC/NA	
11.4	Does the research comply with ethics: anonymity, informed consent, and confidentiality.		Y/PY/PN/N/UC/NA	Yes

Outcome data collection protocol

Evaluation #	Theme (D&D only)	Sub-group (e.g., country)	Measured change (+, 0, -)	Contribution (reported) (Strong/Medium/ Weak/Unclear)	Evidence rating (reported) (Strong/Medium/ Weak/Unclear)	Description (provide page numbers and text)	Outcome category

Note: outcome categories

(1) D&D programmes

- Capacity development activities (or outputs achieved) with partner CSOs
- Capacity development activities (or outputs achieved) with other stakeholders
- Support to L&A activities (or outputs achieved) by partner CSOs
- L&A activities (or outputs achieved) by other stakeholders
- Skills/capacities of local partners/CSOs
- Spillovers to skills/capacities to other local CSOs
- Partnership/coalition building/collaborations with other actors

- L&A activities by local partners/CSOs
- Community-level outcomes
- Policy engagement
- Policy change outcomes
- Policy implementation outcomes

(2) SRHR Programmes

- Activities completed/outputs achieved
- Knowledge/ information
- Girls' attitudes
- Attitudes of other community members
- Girls' empowerment (e.g., involvement in decision making)
- Access to SRHR services
- Access to complementary services
- SRHR service use
- Sexual and reproductive health outcomes

Supplement 2 Assessment results

Table 2.1 Assessment of evaluations of D&D

Evaluation criteria	Study 1	Study 2	Study 3	Study 4	Study 5	Study 6	Study 7	Study 8	Study 9	Study 10	Study 11	Study 12	Study 13	Study 14	Study 15	Study 16	Study 17	Study 18	Study 19	Study 20	Study 21	Study 22	Study 23	Study 24	Study 25	Study 26	Study 27	Study 28
Research Design	High	Medium	Medium	High	Medium	High	Medium	Medium	High	Medium	Medium	High	Medium	High	High	High	Medium	Medium	High	High	High	Medium	Medium	Medium	High	Medium	Medium	High
Methods to evaluate effectiveness	High	Low	Low	High	Medium	Low	Medium	Low	Medium	Low	Low	Medium	Medium	Medium	Low	High	Low	Low	Low	Low	Medium	Low	High	High	Medium	Low	High	High
Theory-based approach	Medium	Low	Low	Medium	Medium	Low	Low	Low	Low	Medium	Medium	Medium	Low	Low	Low	Medium	Low	Low	Low	Low	High	Low	Low	Medium	Low	Low	Low	Medium
Sampling	Low	Low	Low	Medium	Low	Low	Low	Medium	Low	Low	Low	Low	Low	Low	Low	Low	Low	Low	Medium	Low	Low	Medium	Low	Medium	Low	High	Low	Medium
Methods of analysis	High	Low	Low	High	High	Low	Low	High	Medium	Low	Low	Low	Low	Low	Low	High	Medium	Low	Low	Low	Low	High	High	Medium	Medium	High	Medium	Medium
Evaluation matrix	High	Low	Medium	High	Low	Low	Low	High	High	Low	Low	High	Low	Low	Low	Low	Low	Medium	Low	Medium	Low	High	Medium	Medium	Low	Medium	Medium	High
Source of information	High	High	Medium	High	Medium	Low	Low	Medium	Low	High	Low	Medium	Medium	Medium	Low	Low	Medium	Medium	Medium	Medium	Medium	Medium	Low	Low	Medium	Medium	Low	High
Triangulation	High	High	Medium	High	High	Low	Medium	High	Medium	Low	Medium	Low	Low	Low	Low	High	Low	High	High	High	High	High	High	Medium	High	High	High	High
Discussion of bias	Medium	Low	Low	Medium	Low	Low	Low	Medium	Low	Low	Low	Low	Low	Low	Low	Medium	Low	Low	Medium	Low	Low	Low	Low	Low	Low	Low	Low	Medium
Data collection	Medium	Low	Low	Low	Low	Low	Medium	Medium	Low	Low	Low	Low	Low	Medium	Low	High	Medium	Low	Low	High	Low	Low	Medium	Medium	Low	Low	Medium	High
Discussion of limitations	High	Low	Low	High	Low	Medium	High	High	Medium	Low	High	Medium	Low	Low	Low	High	Low	Medium	Medium	Medium	Medium	Medium	High	Medium	Low	Low	High	Medium
**Overall confidence**	**High**	**Low**	**Low**	**High**	**Medium**	**Low**	**Low**	**Medium**	**Low**	**Low**	**Low**	**Medium**	**Low**	**Low**	**Low**	**Medium**	**Low**	**Low**	**Low**	**Medium**	**Medium**	**Medium**	**Medium**	**Medium**	**Medium**	**Medium**	**Medium**	**Medium**

Table 2.2 Assessment of evaluations of SRHR

Evaluation criteria	Study 1	Study 2	Study 3	Study 4	Study 5	Study 6	Study 7	Study 8
Research Design	High	High	High	Low	Low	Medium	Medium	High
Methods to evaluate effectiveness	Medium	Low	Low	Low	Medium	Medium	Low	High
Theory-based approach	Medium	Medium	Low	Low	Medium	Medium	Medium	Medium
Sampling	Low	Medium	Medium	High	Low	Low	Medium	Medium
Methods of analysis	High	Medium	Medium	Low	High	Low	High	High
Evaluation matrix	Low	Low	Low	High	Low	Low	Low	Medium
Source of information	Medium	High	Low	Low	Low	High	Low	Low
Triangulation	High	High	Low	High	Low	High	High	Medium
Discussion of bias	Low	Low	Low	Low	Low	Medium	Low	Low
Data collection	Medium	Medium	Medium	Medium	Medium	Medium	Medium	Low
Discussion of limitations	Low	Medium	High	High	High	High	High	High
**Overall confidence**	**Mediu m**	**Mediu m**	**Low**	**Mediu m**	**Low**	**Mediu m**	**Mediu m**	**Mediu m**
